# Draft Genome Sequence of the Pathogenic Bacterium *Vibrio vulnificus* V252 Biotype 1, Isolated in Israel

**DOI:** 10.1128/genomeA.01182-15

**Published:** 2015-10-15

**Authors:** Vera Efimov, Yael Danin-Poleg, Stefan J. Green, Sharona Elgavish, Yechezkel Kashi

**Affiliations:** a Faculty of Biotechnology and Food Engineering, Technion-Israel Institute of Technology, Haifa, Israel; b Department of Biological Sciences, University of Illinois, Chicago, Illinois, USA; c Info-CORE, Bioinformatics Unit of the I-CORE Computation Center at the Hebrew University and Hadassah, The Institute for Medical Research Israel-Canada, The Hebrew University-Hadassah Medical School, Jerusalem, Israel

## Abstract

We report the genome sequence of the pathogenic *Vibrio vulnificus* biotype 1 clade B, which is suggested to have a common ancestor with biotype 3. This draft genome of the clinical strain V252, isolated in Israel, represents the clonal clade B group that contains both clinical and environmental strains.

## GENOME ANNOUNCEMENT

*Vibrio vulnificus* is an aquatic bacterium and a highly invasive human pathogen (1–4). Strains of *V. vulnificus* are biochemically divided into three biotypes. Biotype 1 is responsible for the majority of human infections worldwide and is a highly varied pathogen (5, 6). Attempts to follow the evolutionary origin of the recently emerged biotype 3 indicate that biotype 3 and the newly identified subgroup clade B of biotype 1, both isolated in Israel, have a common ancestor (7, 8).

Here we describe the draft genome sequence of the clinical *V. vulnificus* biotype 1 clade B strain V252, isolated from a human wound infection in August 2004 at Western Galilee Hospital (collection of the Israeli Ministry of Health 7/04) and subjected to whole-genome shotgun sequencing.

Illumina MiSeq sequencing generated 1,181,041,212 bp (250-bp paired-end reads) with a coverage of 200×. Reads were *de novo* assembled with CLCbio Genomics Workbench version 7.0 (CLC Bio, Denmark) generating 220 contigs >200 bp, with an *N*_50_ of 205,385 bp and the longest contig size of 349,342 bp; 96% of the single reads were mapped to the assembly. There is evidence of the presence of a plasmid related to pYJ016 (contig_9 with high coverage of 620×).

The draft genome of V252 consists of 220 segments covering two chromosomes and one plasmid (5.05Mbp; 46.6% G+C content); 4,383 coding sequences, 68 pseudogenes, 3 rRNAs, 80 tRNAs, and 1 noncoding RNA (ncRNA) were predicted and annotated by the NCBI Prokaryotic Genome Annotation Pipeline (9), similar to the annotation predicted by RAST (10).

Genome alignment of V252 to published *V. vulnificus* genomes, using the NUCmer program of MUMmer version 3.23 (11, 12), revealed higher similarity (89.4%) to *V. vulnificus* VVyb1(BT3) (13) than to YJ016 (85.0%) (14). Comparison using the SEED viewer in RAST (10, 15) revealed that 91.5%, 84.1%, and 78.9% of the V252 genes are similar to VVyb1(BT3), CMCP6, and YJ016, respectively. These findings support the evolutionary relatedness of the V252 and VVyb1(BT3) strains.

Since clade B is highly clonal, the genome of the V252 strain provides representation of this phylogroup, contributing to the understanding of the evolution of this human pathogen.

### Nucleotide sequence accession numbers.

This project has been deposited in DDBJ/EMBL/GenBank under the accession number LIIO00000000. The version described here is the first version, LIIO01000000.
